# Effect of dietary phenylalanine on growth performance and intestinal health of triploid rainbow trout (*Oncorhynchus mykiss*) in low fishmeal diets

**DOI:** 10.3389/fnut.2023.1008822

**Published:** 2023-03-07

**Authors:** Shuze Zhang, Chang’an Wang, Siyuan Liu, Yaling Wang, Shaoxia Lu, Shicheng Han, Haibo Jiang, Hongbai Liu, Yuhong Yang

**Affiliations:** ^1^Key Laboratory of Aquatic Animal Diseases and Immune Technology of Heilongjiang Province, Heilongjiang River Fisheries Research Institute, Chinese Academy of Fishery Sciences, Harbin, China; ^2^College of Animal Science, Northeast Agricultural University, Harbin, China; ^3^College of Life Science, Dalian Ocean University, Dalian, China; ^4^College of Fisheries and Life Science, Shanghai Ocean University, Shanghai, China; ^5^College of Animal Science, Guizhou University, Guiyang, China

**Keywords:** phenylalanine, requirement, triploid *Oncorhynchus mykiss*, intestinal health, low fish meal diet

## Abstract

This study aimed to investigate the effects of phenylalanine on the growth, digestive capacity, antioxidant capability, and intestinal health of triploid rainbow trout (*Oncorhynchus mykiss*) fed a low fish meal diet (15%). Five isonitrogenous and isoenergetic diets with different dietary phenylalanine levels (1.82, 2.03, 2.29, 2.64, and 3.01%) were fed to triplicate groups of 20 fish (initial mean body weight of 36.76 ± 3.13 g). The weight gain rate and specific growth rate were significantly lower (*p* < 0.05) in the 3.01% group. The trypsin activity in the 2.03% group was significantly higher than that in the control group (*p* < 0.05). Amylase activity peaked in the 2.64% treatment group. Serum superoxide dismutase, catalase, and lysozyme had the highest values in the 2.03% treatment group. Liver superoxide dismutase and catalase reached their maximum values in the 2.03% treatment group, and lysozyme had the highest value in the 2.29% treatment group. Malondialdehyde levels in both the liver and serum were at their lowest in the 2.29% treatment group. Interleukin factors IL-1β and IL-6 both reached a minimum in the 2.03% group and were significantly lower than in the control group, while IL-10 reached a maximum in the 2.03% group (*p* < 0.05). The tight junction protein-related genes occludin, claudin-1, and ZO-1 all attained their highest levels in the 2.03% treatment group and were significantly higher compared to the control group (*p* < 0.05). The intestinal villi length and muscle layer thickness were also improved in the 2.03% group (*p* < 0.05). In conclusion, dietary phenylalanine effectively improved the growth, digestion, absorption capacity, antioxidant capacity, and intestinal health of *O. mykiss*. Using a quadratic curve model analysis based on WGR, the dietary phenylalanine requirement of triploid *O. mykiss* fed a low fish meal diet (15%) was 2.13%.

## Introduction

Fish meal is the main protein source for fish feed (1). Due to limited fish meal resources and rising prices, the use of fish meal in fish feed has always been reduced by replacing animal protein sources with plant protein sources in the past decades (2). However, there are some problems, such as nutrient deficiency and indigestion after eating, which hinder the development of fish meal replacement. Fish farming depends on whether the nutritional balance of the feed received by the fish is up to standard (3). Lack of nutrients, especially essential amino acids, can have a serious impact on the growth and health of fish (4). In plant-based feed formulations, critical amino acids including methionine, lysine, and threonine are frequently restricted by amino acids that are frequently added as feed supplements. Plant protein sources may be low in other essential amino acids compared with fish meal (5). Furthermore, fish have lower availability and utilization of plant protein, thereby affecting the availability of essential amino acids in fish. Current practice in the formulation of fish diets is to add methionine, lysine, and threonine to prevent deficiency of essential amino acids (6). Levels of amino acids in the standard were determined and optimized for purified, semi-purified, or fishmeal-based diets and may be insufficient for fish fed a plant-based diet.

Phenylalanine is an aromatic amino acid, which is one of the essential amino acids for fish (7). It is converted to tyrosine in the liver and kidneys, which in turn is a precursor to epinephrine and norepinephrine, thyroid hormone, triiodothyronine, and thyroxine (8). They participate in the functional role of brain chemistry by crossing the blood–brain barrier (9). Tyrosine is also known as a semi-essential amino acid due to the need for additional phenylalanine supplementation to meet the growth and metabolic requirements of fish production when tyrosine levels are insufficient. Aromatic amino acids have an irreplaceable role throughout growth, metabolism, and protein synthesis. It has been determined that a deficiency of phenylalanine in fish will result in decreased feed utilization, reduced antioxidant performance, and impaired growth performance (10, 11). The improvement of phenylalanine in the growth performance of fish may be related to its ability to improve the feed utilization of fish. In previous studies, it was found that the feed utilization rate of aquatic animals such as Indian major carp (*Cirrhinus mrigala*) (12) and catfish *Heteropneustes fossilis* (13) increased with an increase in phenylalanine levels. Molecules like mTOR are able to integrate and regulate the relationship between various nutrients and growth signals in order to regulate the balance between the body’s growth and proliferation rate and the intake of external nutrients. The expression of IGF-1 and mTOR in the hepatopancreas was significantly activated by the addition of phenylalanine to the diet of *Portunus trituberculatus* (14). After the upstream mTOR pathway is activated, the downstream S6K1 and 4EBP-1 genes will also show higher expression levels.

As an important digestive gland, the pancreas secretes a variety of enzymes that can digest protein, lipids, and so on. After the protease passes through the pancreatic duct, it forms trypsin under the action of enterokinase and further activates other proteases. Lipase hydrolyzes glycerides and phospholipids by cutting off lipid bonds (15). Phenylalanine can promote the secretion of protease and bicarbonate in the dog pancreas (16). However, studies in chickens showed that phenylalanine could not promote the secretion of amylase (17). There are few reports on the effects of phenylalanine on the growth and development of fish digestive organs. Only the digestive performance of Jian carp (*Cyprinus carpio* var. Jian) was improved after the phenylalanine supplement (18). Therefore, there may be a positive significance in studying the effect of phenylalanine on the digestive ability of trout.

To resist the damage caused by oxidation, fish also contain antioxidant enzymes, including superoxide dismutase (SOD) and catalase (CAT) (19). Phenylalanine is a precursor of tyrosine, which in turn is a precursor of dopamine and thyroxine. In cultured astrocytes, dopamine increased extracellular SOD protein expression and cell surface SOD activity (20). Thyroxine increased GPx activity and GSH levels in erythrocytes (21). These findings clarify the effects of the antioxidant properties given by phenylalanine to fish. In juvenile carp, it was found that a lack or excess of phenylalanine down-regulated CAT activity, while excess phenylalanine down-regulated SOD gene expression; CAT and SOD gene expression could be up-regulated only when added in appropriate amounts (22). In grass carp (*Ctenopharyngodon idella*), it was shown that 9.57 g/kg of dietary phenylalanine could reduce malondialdehyde (MDA) content in the gills (22).

The intestinal immune barrier in fish is mainly controlled by intestine-associated lymphoid tissues such as monocytes, lymphocytes, macrophages, and granulocytes (23). Nutrients, on the other hand, can modulate the immune system of the intestine by affecting the structural integrity of the intestine (24). In fish, phenylalanine is secreted to produce melanin (25). It was found that melanin can reduce the production of cytokines such as interleukin 1-beta (IL-1β) and interleukin-6 (IL-6) in the body. However, whether dietary phenylalanine has a similar effect on trout has not been reported yet, so whether there is a correlation between phenylalanine and these cytokines deserves further study.

According to the food and agriculture organization of the United Nations (FAO), the annual production of farmed salmon and trout exceeds 3 million tons, making it the third-largest aquaculture species in the world as of 2020 (26). Recently, trout farming in China has developed rapidly and has become one of the main coldwater fish farming species in China, with annual production already reaching 30,000 tons (27). Triploid *Oncorhynchus mykiss* has a faster growth rate, a lower feed coefficient, and a higher meat content than diploid, and it is now the main cultured species of coldwater fish in China (28). The main objective of this study was to investigate the effects of dietary phenylalanine levels on growth performance, intestinal digestive and immune enzyme activity, intestinal gene expression of inflammation and tight junction protein, and the antioxidant capacity of digestive organs of triploid *O. mykiss* fed a low fish meal diet. This will be essential as triploid *O. mykiss* feeds move toward precision formulation.

## Materials and methods

### Feed formulation and preparation

According to the nutritional needs of *O. mykiss*, fish meal and soybean meal were employed as the protein sources, soybean oil and fish oil were used as the sources of lipids, and dextrin was included as the carbohydrate sources. The basic feed with a crude protein level of 41.01% and a crude lipid level of 11.76% was prepared as the control group [phenylalanine level of 1.82% (G1)]. To achieve 2.03% (G2), 2.29% (G3), 2.64% (G4), and 3.01% (G5) phenylalanine levels in the feed, 0.30, 0.60, 0.90, and 1.20% L-phenylalanine (Sigma, 99%) were added, respectively. The tyrosine content was 1.15% (G1), 0.93% (G2), 1.03% (G3), 0.97% (G4), and 1.14% (G5), respectively. Prepare the ingredients according to the formula, and then put them into the mixer and mix well. Ingredients were finely ground before mixing (<250 μm) and then blended with minerals and vitamins. After adding the lipid source, all ingredients were thoroughly mixed for 25 min. Distilled water was then added to achieve the right pellet consistency. The mixture was further homogenized, and a pelletizer (GYJ-250B, Dashiqiao Bao Feed Machinery Factory) was used to form 1-mm pellets. Pellets were dried until the moisture content decreased to about 10% in a ventilated oven at 60°C, and were then stored at −20°C for further use. The formula and nutritional level of the experimental feed are shown in Table 1, and the composition and amount of amino acids in the feed are shown in Table 2.

**TABLE 1:** Experimental diet composition and nutrient levels (air-dry basis, g/kg).

Items	Diets				
	**G1 (1.82%)**	**G2 (2.03%)**	**G3 (2.29%)**	**G4 (2.64%)**	**G5 (3.01%)**
Fish meal^a^	150.00	150.00	150.00	150.00	150.00
Soybean meal^a^	140.00	140.00	140.00	140.00	140.00
Soybean oil^a^	100.00	100.00	100.00	100.00	100.00
Fish oil^a^	62.80	62.80	62.80	62.80	62.80
Compound amino acids^b^	136.80	136.80	136.80	136.80	136.80
Dextrin	200.00	200.00	200.00	200.00	200.00
Gelatin	100.00	100.00	100.00	100.00	100.00
Beer yeast	50.00	50.00	50.00	50.00	50.00
Soybean phospholipid	20.00	20.00	20.00	20.00	20.00
Ca(H_2_PO_4_)_2_	10.00	10.00	10.00	10.00	10.00
Microcrystalline cellulose	10.00	10.00	10.00	10.00	10.00
Vitamin premix^c^	3.00	3.00	3.00	3.00	3.00
Mineral premix^d^	2.00	2.00	2.00	2.00	2.00
Phenylalanine^e^	0.00	3.00	6.00	9.00	12.00
Glycine^e^	12.00	9.00	6.00	3.00	0.00
**Nutrient level^f^**					
Moisture	92.20	92.10	91.80	91.90	92.20
Crude protein	413.20	412.50	409.20	411.40	413.30
Crude lipid	182.80	178.60	176.50	180.40	183.30
Crude ash	37.80	37.30	37.70	37.00	37.50
Gross energy (MJ/kg)	21.73	21.45	21.48	21.62	21.50
Phenylalanine (dry matter)	18.20	20.30	22.90	26.40	30.01
Phenylalanine (protein)	44.39	49.51	55.85	64.38	73.41

^a^Dalong Feed Company, Harbin, China. ^b^Compound amino acids composition: arginine 6.2 g, lysine 21.3 g, phenylalanine 9.0 g, histidine 4.6 g, valine 17.2 g, leucine 20.1 g, isoleucine 14.6 g, cysteine 1.5 g, methionine 6.6 g, proline 8.6 g, aspartic acid 17.8 g, tyrosine 8.2 g, alanine 14.2 g, glutamic acid 36.3 g, and glycine 7.2 g. ^c^The vitamin premix provided the following per kg of diet: VC 100 mg, VE 60 mg, VK_3_ 5 mg, VA 15,000 IU, VD_3_ 3,000 IU, VB_1_ 15 mg, VB_2_ 30 mg, VB_6_ 15 mg, VB_12_ 0.5 mg, nicotinic acid 175 mg, folic acid 5 mg, inositol 1,000 mg, biotin 2.5 mg, and calcium pantothenate 50 mg. ^d^The mineral premix provided the following per kg of diet: MgSO_4_^⋅^7H_2_O 2,000 mg, KCl 1 500 mg, FeSO_4_^⋅^7H_2_O 1,000 mg, CuSO_4_^⋅^5H_2_O 20 mg, MnSO_4_^⋅^4H_2_O 100 mg, ZnSO_4_^⋅^7H_2_O 150 mg, KI 3 mg, NaCl 500 mg, CoCl_2_ 5 mg, and Na_2_SeO_3_ 3 mg. ^e^Sigma Chemical Co., USA. ^f^Nutrient levels were determined values.

**TABLE 2:** Amino acids composition of the basal diets (air-dry basis, %).

Amino acids	Groups				
	**G1 (1.82%)**	**G2 (2.03%)**	**G3 (2.29%)**	**G4 (2.64%)**	**G5 (3.01%)**
**Non-essential amino acid**					
Asp	1.90	2.51	3.03	2.77	2.79
Ser	1.40	1.08	1.36	1.25	1.71
Glu	5.27	5.26	5.20	4.73	5.45
Gly	2.40	2.98	2.34	1.78	2.04
Ala	2.53	2.60	3.15	2.98	2.84
Cys	0.76	0.89	0.85	0.91	0.69
Tyr	1.15	0.93	1.03	0.97	1.14
Pro	2.89	2.43	2.65	2.79	3.14
**Essential amino acid**					
Thr	1.19	1.00	1.28	1.16	1.59
Val	1.74	1.28	1.56	1.43	1.90
Met	1.31	1.76	2.05	2.23	2.18
Ile	1.23	0.84	1.04	0.95	1.27
Leu	4.20	3.68	3.35	3.03	3.27
Phe	1.82	2.03	2.29	2.64	3.01
Lys	2.26	1.64	2.10	1.93	1.60
His	0.82	1.18	0.97	0.89	1.22
Arg	1.95	1.40	1.91	1.76	1.93
TAA	34.82	33.49	36.16	34.20	34.76

Asp, aspartate; Thr, threonine; Ser, serine; Glu, glutamate; Gly, glycine; Ala, alanine; Cys, cysteine; Val, valine; Met, methionine; Ile, isoleucine; Leu, leucine; Tyr, tyrosine; Phe, phenylalanine; Lys, lysine; His, histidine; Arg, arginine; Pro, proline; EAA, essential amino acid; TAA, total amino acids.

### Feeding trial

Triploid *O. mykiss* was purchased from Egremorin Industries (Benxi, China) and acclimated for 15 days. Control diets were fed throughout the acclimate period. Before the feeding experiment, a total of 300 fish with an initial average weight of (36.76 ± 3.13 g) were allocated to 15 tanks, with 20 healthy and uniform fish per replicate, and three replicates per treatment group. The experiment was carried out in an indoor aquarium with a controlled water circulation system. A feeding trial was conducted for 8 weeks, during which the fish were fed the test diets twice daily, at 9:00 a.m. and 4:00 p.m., until satiation.

Feeding condition: the water source was aerated tap water. Water temperature was maintained at 14 ± 0.5°C. The water dissolved oxygen concentration is >6.0 mg/L, NO_2_^–^-N < 0.02 mg/L, pH 6.8–7.1, and NH_4_^+^-N < 0.2 mg/L, respectively. Water quality parameters were measured using a YSI-556 multi-parameter water quality meter (YSI Inc., Yellow Springs, OH, USA). One-third of the water is changed every afternoon to ensure water clarity and sufficient dissolved oxygen.

### Sample collection

At the end of the experiment, fish were starved for 24 h to allow emptying of the digestive tract contents prior to sampling. All fish were weighed to calculate weight gain rate and other growth indicators [ME204E, Mettler-Toledo Technologies (China) Co.]. Nine fish were randomly selected from each treatment group and anesthetized with tricaine methanesulfonate MS-222 (75 mg/L). Blood samples were obtained from the tail vein, then centrifuged at 4,000 × *g* for 10 min at 4°C, and the supernatant was extracted as serum. The serum was stored at −20°C for subsequent serum biochemical assays. The mid-intestines of three fish were stored at −40°C for biochemical analyses. The intestines of the other three fish were removed and immediately frozen in liquid nitrogen and stored at −80°C at the end of sampling for subsequent gene expression assays. The other three fish intestines were stored in Bouin’s solution for future histomorphological observation.

### Nutritional content

The experimental diets and fish were analyzed using an AOAC-based protocol (29). Moisture content was determined by drying the samples in an oven at 105°C until a constant weight was obtained. Crude protein (N × 6.25) was analyzed by measuring nitrogen using the Kjeldahl method (2300, FOSS, Sweden). Ash content was analyzed by carbonization at 300°C for 30 min, followed by incineration at 550°C for 4 h. Crude lipid was measured by the Soxhlet method (Extraction System-811, BUCHI, Switzerland).

### Amino acid determination

Before the start of amino acid determination of fish and feed, 40–50 mg (accurate to 0.1 mg) of the sample was weighed with an electronic analytical balance, and 10 ml of hydrochloric acid with a concentration of 6 mol/L was added. The ampoule was then heated by an alcoholic blowtorch and sealed immediately, and then placed in a constant temperature oven for 22 h of hydrolysis, setting the temperature at 110°C. After cooling, 10 ml of 6 mol/L sodium hydroxide solution was added to the alkali neutralization. Then the solution was poured into a 100 ml volumetric flask, fixed with 0.02 mol/L hydrochloric acid, and mixed well with the sample hydrolysis solution. The sample was filtered through a 0.2 μm filter membrane into the sample bottle before the machine, and then was determined by an automatic amino acid analyzer (L-8900, Hitachi, Japan).

### Biochemical analysis

Biochemical analysis assays were performed using commercially available kits according to the manufacturer’s protocol (Nanjing Jiancheng Institute of Biological Engineering, Nanjing, China). CAT (A007-2-1) activity was determined by measuring the decrease in H_2_O_2_ concentration at 240 nm. The reaction mixture contained 50 mm of potassium phosphate buffer (pH 7.0) and 10.6 mM of freshly prepared H_2_O_2_. SOD) (A001-3-2) activity was measured spectrophotometrically using xanthine/xanthine oxidase as a source of superoxide radicals. The reaction mixture consisted of 50 mM potassium phosphate buffer (pH 7.8), 0.1 mM EDTA, 0.1 mM xanthine, 0.013 mM cytochrome c, and 0.024 IU/ml xanthine oxidase. An activity unit was defined as the amount of enzyme required to produce 50% inhibition of the rate of reduction of ferrocyanic measured at 550 nm. The amount of lysozyme (LZM; A050-1-1) was measured by a turbidimetric assay. By destroying the β-1,4-glycosidic bond between n-acetyl acetylmuramic acid and n-acetyl glucosaccharide in the cell wall, the cell wall insoluble monosaccharide is decomposed into soluble glycopeptides, resulting in the rupture of the cell wall and the escape of the contents to make the bacteria dissolve. Lipid peroxidation was analyzed in MDA (A003-1-2) equivalents using a thiobarbituric acid reaction. The reaction was carried out at a colorimetric wavelength of 532 nm.

Homogenized intestinal samples were centrifuged at 6,000 × *g* for 20 min at 4°C in 10 volumes (w/v) of ice-cold saline. Subsequently, the supernatant was used for biochemical analysis using a lipase assay kit (LPS; A054-2-1) (30) and an amylase assay kit (AMS; C016-1-1) (31). Trypsin (A080-2-2) (32) content was determined by the UV colorimetric method; amylase (AMS) activity was determined by the starch iodine colorimetric method; lipase (LPS) content was determined by the colorimetric method, and protein content was determined by the Thomas Brilliant Blue method (33, 34). All kits were purchased from Nanjing Jiancheng Reagent Company and used according to the instructions.

### Histological examination

The mid-intestines of three fish in each replicate were randomly fixed in Bouin’s solution for 48 h, then washed several times with water to remove the fixative, and embedded by conventional paraffin immersion. A microtome (KD 1508) was used to cut sections to a thickness of 6 μm. Sections were successively destained with ethanol, stained with hematoxylin and eosin, and finally sealed with neutral resin. After observation with a microscope (Leica MD 4000B), there were more than 10 intestinal slices in each group.

### Real-time quantitative PCR

Total RNA was isolated from intestinal tissues using RNAiso Plus (TaKaRa, China). The quality of the RNA was determined by analyzing the integrity of the RNA by agarose gel electrophoresis and confirming the absorbance ratio at A260/A280 nm between 1.8 and 2.0. The proposed RNA was reverse transcribed to cDNA using the PrimeScript™ RT reagent kit (TaKaRa, Dalian, China) and stored at −80°C in the refrigerator until use. Quantitative PCR (qPCR) was performed on a LightCycler^®^ 480 thermal cycler (Roche, Germany) in a total volume of 10 ml using a Light Cycler^®^ 480 SYBR Green I Master (Roche, Germany), following the manufacturer’s protocol. All amplification reactions were compared using three replicates. All primer sequences in this experiment were referenced to the primer sequence of the *O. mykiss* gene published by Lee et al. (30) and Evenhuis et al. (35), as detailed in Table 3. β-Actin was used as an internal reference gene for the normalization of cDNA loading (36). The cycling conditions were 95°C for 30 s followed by 35 cycles of 95°C for 5 s, 59°C for 10 s, and 72°C for 30 s. Expression results were analyzed by the 2^–ΔΔ^ ^CT^ method.

**TABLE 3:** Primer sequences used for gene expression analyses.

Genes	Primer sequences forward (5′-3′)	Primer sequences reverse (5′-3′)	Amplicon (bp)	Accession number	Primer efficiency (%)
β-Actin	F: GCCGGCCGCGACCTCACAGACTAC	R: CGGCCGTGGTGGTGAAGCTGTAAC	73	AC00648	99.65
IL-1β	F: CTCTACCTGTCCTGCTCCAAA	R: ATGTCCGTGCTGATGAACC	194	AB010701.1	92.00
IL-6	F: CAATCAACCCTACTCCCCTCT	R: CCTCCACTACCTCAGCAACC	91	FR715329	96.00
IL-2	F: AGAATGTCAGCCAGCCTTGT	R: TCTCAGACTCATCCCCTCAGT	69	NM_001124657.1	95.00
IL-10	F: CGACTTTAAATCTCCCATCGAC	R: GCATTGGACGATCTCTTTCTTC	70	AB118099.1	96.00
TNF-α	F: CCACACACTGGGCTCTTCTT	R: GTCCGAATAGCGGGAAATAA	128	AJ278085.1	96.00
TGF-β	F: TCCGCTTCAAAATATCAGGG	R: TGATGGCATTTTCATGGCTA	71	AJ007836.1	95.00
NF-κB	F: CAGGACCGCAACATACTGGA	R: GCTGCTTCCTCTGTTGTTCCA	92	XM_031794907.1	95.00
Claudin-1	F: TAGCATCCACGATCA	R: GAGCCTTCACTGGAGC	124	BK00876	96.00
ZO-1	F: CTGCTGGACGAAGGGA	R: GGCCTTTATCCTGCAT	191	HQ6560	95.00
Occludin	F: ATGGCTCAATCTACAGG	R: GAGATACTGGTTGACCAACC	102	FR904483.1	95.00
TOR	F: CCAAAGAGATGCAGAAGCCACA	R: CTCTCTCATACGCTCTCCCT	178	XM_020506200.2	98.00
IGF-1	F: ACTGTGCCCCTGCAAGTCT	R: CTGTGCTGTCCTACGCTCTG	159	M81904	93.00
GH	F: CAAAGTGGGCATCAA	R: GTTCCTCCTGACGT	139	NM_0011246	96.00
GHR	F: TCCCCTTCACCAGGA	R: TCATTCTGCAGTGGC	148	AB10083	97.00
S6K1	F: CCTCCTCATGACACCCTGCT	R: TCTTCTGGTCCGTTGGCAAA	129	XM_029674978.1	94.00
4EBP-1	F: GGGGAACTCTGTTCAGCACA	R: AATGTTGGGGAGAGAGCACG	117	NM_004095	94.00

IL-1β, interleukin-1β; IL-6, interleukin-6; IL-2, interleukin-2; IL-10, interleukin-10; TNF-α, tumor necrosis factor-α; TGF-β, transforming growth factor-β; NF-κB, nuclear factor-κB; S6K1, S6 kinase 1; IGF-1, insulin growth factor 1; GH, growth hormone; GHR, growth hormone receptor; S6K1, 4EBP-1, eukaryotic initiation factor 4E-binding proteins-1.

### Calculation formula of growth performance

Weight gain rate (WGR; %) = 100 × (W_t_ − W_0_) / W_0_;
Condition factor (CF; %) = 100 × W_t_ / Lt^3^;
Feed conversion ratio (FCR) = W_f_ / (W_t_ − W_0_);
Specific growth rate (SGR, %/d) = 100 × (lnW_t_ − lnW_0_) / t;
Hepatosomatic index (HSI; %) = 100 × (liver weight (g) / body weight (g));
Viscerosomatic index (VSI; %) = 100 × (viscera (g) / body weight (g)).

W_0_ is the initial body mass of the fish (g); W_t_ is the terminal body mass (g); L_t_ is the terminal body length of the fish (cm); W_f_ is the feed intake (g); T is the test day (d) (37).

Statistical software SPSS 20.0 for Windows (SPSS Inc., Chicago, IL, USA) was used to conduct the one-way analysis of variance and Duncan’s multiple comparisons of the data. All data were expressed as mean ± standard error (SE), with *p* < 0.05 used as the significant difference standard (36). The quadratic regression analysis of significant difference indices was carried out by Graphpad Prism 8.0 to determine the optimal demand range of phenylalanine for triploid *O. mykiss* under the condition of low fish meal (38). The bar charts in the article were also plotted using Graphpad Prism 8.0.

## Results

### Growth performance and somatic indices

**FIGURE 1:**
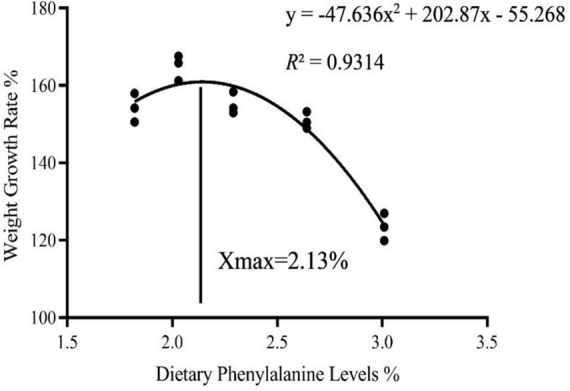
Quadratic regression analysis based on the weight gain rate of triploid *O. mykiss* fed experimental diets for 8 weeks.

**FIGURE 2:**
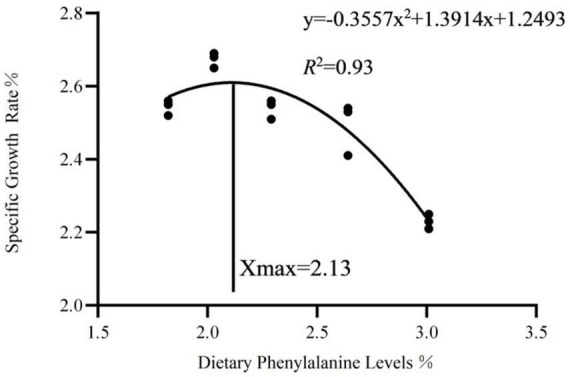
Quadratic regression analysis based on the specific growth rate of triploid *O. mykiss* fed experimental diets for 8 weeks.

As dietary phenylalanine levels increased, the WGR and SGR of triploid *O. mykiss* increased and then decreased, reaching a maximum in the 2.03% group and being significantly higher than the 3.01% group (*p* < 0.05) and the VSI ratio in the 2.03% group being significantly higher in all groups (*p* < 0.05) (Table 4). Primary, secondary, and tertiary linear regression equations were analyzed for WGR and SGR of triploid *O. mykiss* to determine the optimal addition of phenylalanine under low fishmeal feed conditions (Table 5). By comparing the *R*^2^ values, the quadratic equation provided good fits. From the regression analysis, it was shown that the WGR of triploid *O. mykiss* had a significant quadratic response to the increase in phenylalanine levels in the diet. The optimal phenylalanine requirement for triploid *O. mykiss* based on WGR was estimated to be 2.13% (Figures 1, 2).

**TABLE 4:** Effects of dietary phenylalanine levels on the growth performance and somatic indices of triploid *O. mykiss*.

Items	Groups					
	**G1 (1.82%)**	**G2 (2.03%)**	**G3 (2.29%)**	**G4 (2.64%)**	**G5 (3.01%)**	**SE**
Initial body weight/g	35.15 ± 1.72	39.09 ± 1.00	37.52 ± 3.92	36.87 ± 5.23	35.15 ± 3.78	0.04
Final body weight/g	89.33 ± 2.58^b^	103.90 ± 1.83^d^	95.37 ± 3.04^c^	92.35 ± 3.83^bc^	78.53 ± 1.57^a^	1.23
WGR/%	154.14 ± 3.35^ab^	165.80 ± 4.37^b^	154.18 ± 6.92^ab^	150.47 ± 5.51^ab^	123.41 ± 4.46^a^	1.53
FCR	1.35 ± 0.18^b^	1.25 ± 0.31^a^	1.50 ± 0.15^c^	1.45 ± 0.23^d^	2.19 ± 0.55^e^	0.45
SGR/(%/d)	2.54 ± 0.06^ab^	2.66 ± 0.04^b^	2.54 ± 0.07^ab^	2.50 ± 0.06^ab^	2.23 ± 0.04^a^	0.02
SR (%)	96.00 ± 4.00	96.00 ± 6.93	96.00 ± 6.93	93.33 ± 6.11	93.33 ± 8.33	1.02
CF	1.22 ± 0.09	1.23 ± 0.12	1.19 ± 0.03	1.16 ± 0.10	1.14 ± 0.09	0.03
HIS (%)	1.48 ± 0.59^a^	2.12 ± 0.51^c^	1.95 ± 0.41^bc^	1.92 ± 0.36^bc^	1.67 ± 0.27^ab^	0.14
VSI (%)	11.49 ± 1.94^a^	13.40 ± 1.79^b^	12.76 ± 1.89^ab^	13.39 ± 1.44^b^	13.49 ± 1.33^b^	0.24

Values are presented as mean ± SE (*n* = 3). Values in the same column with different superscript letters are significantly different (*p* < 0.05). WGR, weight gain rate; FCR, feed conversion rate; SGR, specific growth rate; PER, protein efficiency ratio; CF, condition factor; SR, survival rate; HSI, hepatosomatic index; VSI, viscerosomatic index.

**TABLE 5:** Linear modeling of the effect of dietary phenylalanine to low fish meal diets on weight gain rate and specific growth rate of triploid *O. mykiss*.

	Primary folding line model	Quadratic linear model	Cubic linear model
WGR	y = −39.398x + 246.47	y = −47.636x^2^ + 202.87x–55.268	y = 6.4319x^3^–93.522x^2^ + 309.85x–136.76
*R* ^2^	*R*^2^ = 0.9061	*R*^2^ = 0.9314	*R*^2^ = 0.9061
SGR	y = −0.4074x + 3.498	y = −0.3557x^2^ + 1.3914x + 1.2493	y = 0.1016x^3^–1.2126x^2^ + 3.7717x–0.9027
*R* ^2^	*R*^2^ = 0.914	*R*^2^ = 0.93	*R*^2^ = 0.931

Equations and R^2^ were calculated based on weight gain rates and specific growth rates, and data statistics were obtained from PRISM 8.

### Effects of dietary phenylalanine levels on nutritional composition in triploid *O. mykiss* fed low fish meal diets

Whole-body crude protein levels peaked in the 2.03% group, which was significantly higher than the control group (*p* < 0.05). The highest whole fish lipid content was obtained when fed 2.03% phenylalanine and was significantly different from the other groups (*p* < 0.05) (Table 6). Meanwhile, dietary phenylalanine levels did not significantly affect the moisture and crude ash composition of triploid *O. mykiss* whole fish.

**TABLE 6:** Effects of dietary phenylalanine levels on the body composition of triploid *O. mykiss*.

Indices	Groups					
	**G1 (1.82%)**	**G2 (2.03%)**	**G3 (2.29%)**	**G4 (2.64%)**	**G5 (3.01%)**	**SE**
Moisture	70.01 ± 1.37	68.72 ± 1.78	68.78 ± 0.76	69.62 ± 0.65	69.30 ± 1.76	0.03
Crude protein	14.21 ± 1.50^a^	15.28 ± 2.99^b^	14.68 ± 2.97^ab^	14.30 ± 1.56^a^	13.17 ± 1.38^a^	0.21
Crude lipid	10.46 ± 1.15^a^	10.93 ± 1.14^b^	10.71 ± 2.63^a^	10.67 ± 0.79^a^	10.52 ± 1.88^a^	0.16
Ash	2.38 ± 0.02	2.50 ± 0.09	2.48 ± 0.13	2.49 ± 0.07	2.35 ± 0.04	0.04

Values are presented as mean ± SE (*n* = 3). Values in the same column with different superscript letters are significantly different (*p* < 0.05).

### Effects of dietary phenylalanine levels on amino acid composition in triploid *O. mykiss* fed low fish meal diets

Under low fish meal feed conditions, dietary phenylalanine levels significantly affected the amino acid profile (*p* > 0.05), except for valine (*p* < 0.05). Dietary phenylalanine had no significant effect (*p* > 0.05) on the levels of the first limiting amino acid, methionine, and the second limiting amino acid, lysine (Table 7). The tyrosine content at the end of the experiment was 1.78% (G1), 1.77% (G2), 1.71% (G3), 1.79% (G4), and 1.76% (G5), respectively. There was no significant difference between the treatment groups (*p* > 0.05).

**TABLE 7:** Effects of dietary phenylalanine levels on the amino acid composition of whole triploid *O. mykiss*.

Indices	Groups					
	**G1 (1.82%)**	**G2 (2.03%)**	**G3 (2.29%)**	**G4 (2.64%)**	**G5 (3.01%)**	**SE**
**Essential amino acid**						
Thr	2.68 ± 0.32	2.64 ± 0.11	2.28 ± 0.18	2.70 ± 0.08	2.68 ± 0.30	0.32
Val	2.79 ± 0.16^ab^	2.76 ± 0.20^ab^	2.39 ± 0.23^a^	2.93 ± 0.19^b^	2.59 ± 0.27^ab^	0.03
Met	2.11 ± 0.01	2.10 ± 0.01	2.10 ± 0.01	2.11 ± 0.01	2.10 ± 0.01	0.04
Ile	2.47 ± 0.10	2.47 ± 0.16	2.29 ± 0.21	2.63 ± 0.14	2.39 ± 0.23	0.17
Leu	6.46 ± 0.25	6.36 ± 0.39	5.90 ± 0.71	6.56 ± 0.33	6.08 ± 0.68	0.36
Phe	2.41 ± 0.02^a^	2.57 ± 0.19^a^	2.79 ± 0.09^b^	2.84 ± 0.12^b^	3.03 ± 0.09^c^	0.02
Lys	8.05 ± 0.34	8.13 ± 0.23	7.84 ± 0.72	8.26 ± 0.26	8.36 ± 0.01	0.13
His	1.38 ± 0.20	1.19 ± 0.16	1.21 ± 0.20	1.28 ± 0.15	1.11 ± 0.13	0.32
Arg	4.25 ± 0.35	3.96 ± 0.27	3.79 ± 0.58	4.17 ± 0.29	3.83 ± 0.40	0.07
**Non-essential amino acid**						
Asp	9.03 ± 0.54	9.34 ± 0.23	9.29 ± 0.01	9.35 ± 0.22	9.33 ± 0.37	0.27
Ser	2.88 ± 0.29^b^	2.64 ± 0.41^b^	2.05 ± 0.30^a^	2.65 ± 0.19^b^	2.32 ± 0.26^ab^	0.25
Glu	10.83 ± 0.14^b^	10.72 ± 0.08^ab^	10.59 ± 0.13^a^	10.77 ± 0.09^ab^	10.63 ± 0.08^ab^	0.43
Gly	4.20 ± 0.65^b^	3.58 ± 0.28^ab^	3.24 ± 0.41^a^	3.76 ± 0.38^ab^	3.29 ± 0.37^a^	0.06
Ala	4.24 ± 0.33	4.08 ± 0.01	4.15 ± 0.12	4.37 ± 0.25	4.10 ± 0.01	0.24
Cys	1.16 ± 0.03^b^	0.88 ± 0.08^a^	0.91 ± 0.09^a^	1.10 ± 0.12^b^	0.88 ± 0.08^a^	0.04
Tyr	1.78 ± 0.04	1.77 ± 0.13	1.71 ± 0.23	1.79 ± 0.08	1.76 ± 0.12	0.13
Pro	2.66 ± 0.21^b^	2.19 ± 0.29^ab^	2.02 ± 0.38^a^	2.26 ± 0.25^ab^	1.90 ± 0.25^a^	0.37
Total	69.57 ± 2.57	67.17 ± 2.68	64.19 ± 3.79	69.20 ± 3.10	65.32 ± 3.75	0.21

Values are presented as mean ± SE (*n* = 3). Values in the same column with different superscript letters are significantly different (*p* < 0.05).

### Effects of dietary phenylalanine levels on the antioxidant capacity in triploid *O. mykiss* fed low fish meal diets

The effects of dietary phenylalanine on antioxidant parameters in the serum and liver are displayed in Table 8. The serum SOD reached a maximum in the 2.03% group and was significantly higher than the control group (*p* < 0.05). There was no significant difference in liver SOD among different treatment groups (*p* > 0.05). Liver CAT peaked in the 2.03% treatment group and was significantly higher than in the other treatment groups (*p* < 0.05). There was no significant difference in serum MDA among treatment groups (*p* > 0.05), while liver MDA showed a trend of increasing and then stabilizing, reaching the maximum in the 2.29% group (*p* < 0.05). Serum and liver LZM reached the highest values at 2.03 and 2.29% of phenylalanine content, respectively, and were significantly different compared to the control group (*p* < 0.05).

**TABLE 8:** Effects of dietary phenylalanine levels on the antioxidant capacity of triploid *O. mykiss*.

Indices	Groups					
	**G1 (1.82%)**	**G2 (2.03%)**	**G3 (2.29%)**	**G4 (2.64%)**	**G5 (3.01%)**	**SE**
SOD (U/mL)						
Serum	39.65 ± 3.62^b^	46.48 ± 6.24^c^	34.76 ± 2.89^b^	36.83 ± 3.16^b^	27.84 ± 1.93^a^	1.25
Liver	10.22 ± 1.58	12.85 ± 2.59	10.94 ± 2.66	11.10 ± 2.92	12.76 ± 1.09	0.07
**CAT (U/mL)**						
Serum	21.30 ± 11.60^a^	37.51 ± 18.22^b^	24.74 ± 5.53^a^	32.30 ± 7.70^b^	33.78 ± 12.13^b^	1.56
Liver	17.33 ± 1.23^a^	21.98 ± 3.34^b^	17.59 ± 2.98^a^	17.85 ± 4.46^a^	21.35 ± 2.33^b^	1.32
**MDA (nmol/mL)**						
Serum	2.00 ± 0.15	1.76 ± 0.23	1.66 ± 0.44	2.00 ± 0.51	2.12 ± 0.40	0.11
Liver	5.15 ± 0.88^b^	3.85 ± 0.64^a^	3.56 ± 0.33^a^	3.81 ± 0.14^a^	3.94 ± 0.22^a^	0.32
**LZM (U/mL)**						
Serum	75.84 ± 7.32^a^	94.67 ± 11.00^b^	93.27 ± 7.21^b^	73.12 ± 8.01^a^	64.78 ± 8.14^a^	0.44
Liver	20.05 ± 8.40^a^	40.36 ± 2.14^b^	54.67 ± 8.84^c^	44.19 ± 8.37^bc^	22.95 ± 6.48^a^	1.89

Values are presented as mean ± SE (*n* = 3). Values in the same column with different superscript letters are significantly different (*p* < 0.05). SOD, superoxide dismutase; MDA, malondialdehyde; CAT, catalase; LAZ, lysozyme.

### Effects of dietary phenylalanine levels on the intestinal digestive enzyme in triploid *O. mykiss* fed low fish meal diets

The effects of different dietary phenylalanine levels on the intestinal digestive enzyme activity of triploid *O. mykiss* are shown in Table 9. Trypsin activity was significantly higher in the 2.03% group than in the control group (*p* < 0.05). The AMS activity in the 2.03 and 2.29% groups was significantly higher than that in the control and other treatment groups (*p* < 0.05), but showed a gradual decrease with the increase in phenylalanine level. Dietary phenylalanine levels had no significant effect on the LPS activity of triploid *O. mykiss* (*p* > 0.05).

**TABLE 9:** Effects of dietary phenylalanine levels on the digestive enzyme of triploid *O. mykiss*.

Indices	Groups					
	**G1 (1.82%)**	**G2 (2.03%)**	**G3 (2.29%)**	**G4 (2.64%)**	**G5 (3.01%)**	**SE**
Trypsin (U/mgprot)	1,716.59 ± 685.53^a^	4,602.81 ± 717.13^c^	3,006.85 ± 687.26^b^	2,874.80 ± 650.82^b^	2,900.62 ± 778.87^b^	78.76
Lipase (U/gprot)	43.39 ± 13.27	43.43 ± 11.51	46.07 ± 6.79	48.67 ± 8.55	44.55 ± 6.32	2.76
Amylase (U/gprot)	72.38 ± 6.24^a^	106.82 ± 12.06^b^	176.07 ± 21.17^c^	85.03 ± 3.93^ab^	92.56 ± 12.41^ab^	8.97

Values are presented as mean ± SE (*n* = 3). Values in the same column with different superscript letters are significantly different (*p* < 0.05).

### Effects of dietary phenylalanine levels on the intestinal tissue morphology of triploid *O. mykiss*

**FIGURE 3:**
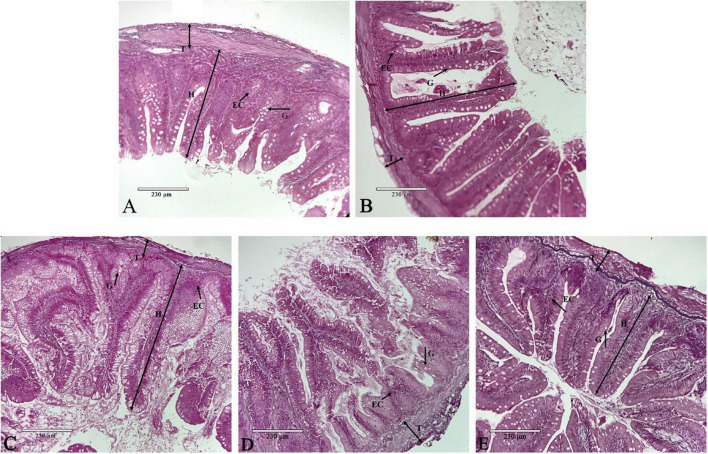
Intestine histology. Representative histological sections of the intestine from *O. mykiss* fed the different experimental diets. Scale bar, 230 μm. Panel **(A)** was G1 phenylalanine level in intestine tissue 100×; Panel **(B)** was G2 phenylalanine level in intestine tissue 100×; Panel **(C)** was G3 phenylalanine level in intestine tissue 100×; Panel **(D)** was G4 phenylalanine level in intestine tissue 100×; and panel **(E)** was G5 phenylalanine level in intestine tissue 100×.

Dietary phenylalanine levels had significant effects on the structural morphology of the intestine of *O. mykiss*. In Figure 3A (1.82% group), the intestinal villi were neatly arranged, and the surface striate margin was smooth. In Figure 3B (2.03% group), the length of the villi was longer and there were more cup-shaped cells and epithelial cells. In Figure 3C (2.29% group), the length of the villi reached its longest length and was significantly higher than the other treatment groups. However, the thickness of the muscle layer was thinner than that of the second group. In Figure 3D (2.64% group), the nucleus shift phenomenon began to appear, and the apical part of the villi started to shed. In Figure 3E (3.01% group), the intestinal muscular thickness was significantly lower, and the nuclei of the epithelial cells shifted significantly.

The length of the villi and the thickness of the muscular layer are shown in Table 10. Villi length reached a maximum in the 2.29% treatment group and was significantly higher than that in the other treatment groups (*p* < 0.05). The thickness of the muscular layer was significantly higher in the 2.03% treatment group than that in the control group (*p* < 0.05).

**TABLE 10:** Effects of dietary phenylalanine levels on the intestinal morphology of triploid *O. mykiss* (μm).

Indices	Groups					
	**G1 (1.82%)**	**G2 (2.03%)**	**G3 (2.29%)**	**G4 (2.64%)**	**G5 (3.01%)**	**SE**
Villus length	442.44 ± 3.30^a^	511.88 ± 18.31^b^	684.44 ± 4.26^c^	464.97 ± 36.59^ab^	465.11 ± 15.93^ab^	8.86
Muscular layer thickness	88.15 ± 5.38^b^	101.22 ± 7.56^b^	61.53 ± 4.17^a^	67.88 ± 0.94^a^	67.55 ± 8.38^a^	2.64

Values are presented as mean ± SE (*n* = 3). Values in the same column with different superscript letters are significantly different (*p* < 0.05).

### Expression of IGF-1, GH, GHR, TOR, S6K1, and 4EBP-1 in the intestine of *O. mykiss*

**FIGURE 4:**
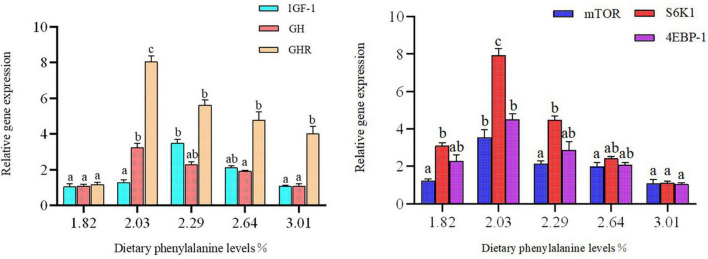
Growth gene expression of the intestine. Lowercase letters (a, b, or c) indicate a significant effect of liver growth on gene expression (*p* < 0.05). GH, growth hormone; IGF-1, insulin growth factor 1; GHR, growth hormone receptor; 4E-BP, eukaryotic initiation factor 4E-binding proteins; S6K1, S6 kinase 1.

Dietary phenylalanine levels significantly affected the expression of intestinal growth-related genes in triploid *O. mykiss* (*p* < 0.05) (Figure 4). The expression levels of mTOR, downstream S6K1, and 4EBP-1 genes in the 2.03% treatment group reached their highest values, and there were significant differences with the 3.01% treatment group (*p* < 0.05). Similarly, GHR and GH gene expression levels were all highest in the 2.03% treatment group and significantly higher than the control group (*p* < 0.05).

### Expression of cytokines IL-1β, IL-2, IL-6, IL-10, TGF-β, TNF-α, and NF-κB in the intestine of *O. mykiss*

**FIGURE 5:**
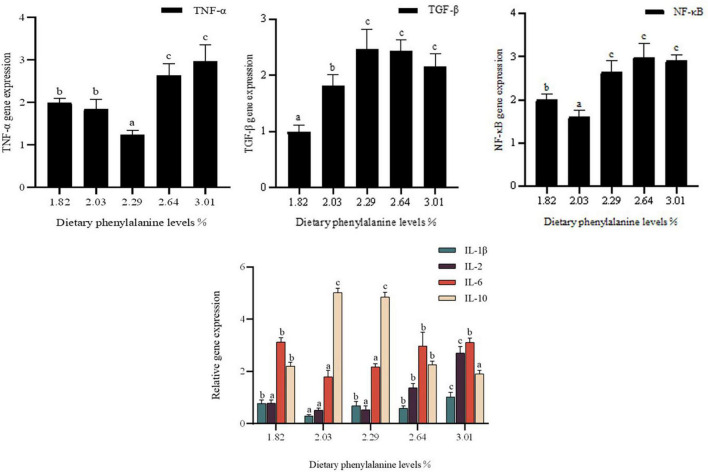
Immunity gene expression of the intestine. Lowercase letters (a, b, or c) indicate a significant effect of immune-related gene expression in the intestine (*p* < 0.05). IL-1β, interleukin-1β; IL-2, interleukin-2; IL-6, interleukin-6; IL-10, interleukin-10; TNF-α, tumor necrosis factor-α; TGF-β, transforming growth factor-β; NF-κB, nuclear transcription factors.

Dietary phenylalanine levels significantly affected the expression of interleukin (IL-1β, IL-2, IL-6, and IL-10) genes, TGF-β, and TNF-α in the intestine of triploid *O. mykiss* (*p* < 0.05) (Figure 5). IL-1β gene expression reached a minimum at a phenylalanine level of 2.03%, which was significantly lower than in the control group (*p* < 0.05). The expression of pro-inflammatory factors IL-2 and IL-6 was lowest in the 2.29 and 2.03% treatment groups, respectively, which was significantly different from the 3.01% treatment group (*p* < 0.05).

In triploid *O. mykiss* fed low fish meal diets, dietary phenylalanine levels had a significant effect on the expression of intestinal tumor necrosis factor (TNF-α) and nuclear factor-κB (NF-κB) genes (*p* < 0.05). TNF-β gene expression was lower in the 2.29% treatment group than in the control group (*p* < 0.05). TGF-β gene expression was highest in the 2.29% treatment group. TGF-β gene expression reached a maximum in the 2.29% treatment group. The nuclear transcription factor NF-κB also differed significantly among the groups. Compared to the other treatment groups, 2.03% of the treatment groups had significantly lower NF-κB mRNA expression (*p* < 0.05), while there was no significant difference among the G3–G5 groups.

### Effects of dietary phenylalanine levels on intestinal tight junction protein-related genes in triploid *O. mykiss* fed low fish meal diets

**FIGURE 6:**
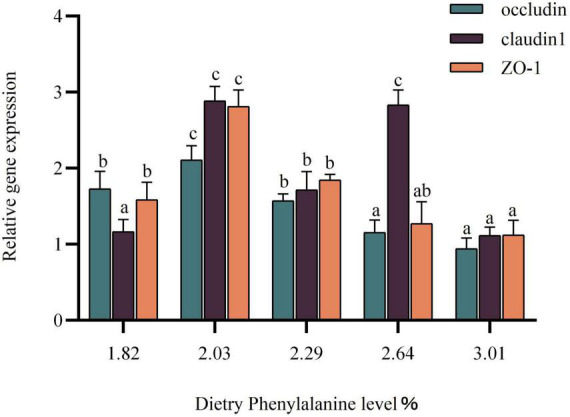
Tight junction protein gene expression of the intestine. Lowercase letters (a, b, or c) indicate significant effects of intestinal tight junction proteins relative to gene expression (*p* < 0.05).

The expression of the intestinal tight junction protein gene was gradually increased as dietary phenylalanine levels ranged from 1.82 to 2.29%. The occludin gene in triploid *O. mykiss* showed a trend of increasing and then decreasing compared with the control group (*p* < 0.05) (Figure 6). The claudin-1 gene reached a maximum in the 2.03% groups and was significantly higher than that in the other groups (*p* < 0.05). The ZO-1 gene expression peaked in the 2.03% group, which was significantly different from the control group (*p* < 0.05).

## Discussion

### Effects of phenylalanine levels on the growth performance of triploid *O. mykiss*

Phenylalanine is an EAA for protein synthesis and growth stimulation in fish. Dietary phenylalanine can enhance fish feeding and increase the WGR and SGR of fish. This study showed that in low fish meal diets (phenylalanine level of 1.82%), the WGR and SGR of triploid *O. mykiss* showed a trend of increasing and then decreasing with increasing phenylalanine levels. Similar results were observed in Indian major carp and silver perch (12, 39, 40). In pomfrets (*Pampus punctatissimus*), it was found that the lack or excess of phenylalanine in the diet would lead to reduced growth performance and feed conversion rate (41). Phenylalanine deficiency and excess will disrupt the amino acid balance of the feed. The balance of amino acids in the feed will be disrupted, affecting the absorption and utilization of amino acids in the feed by fish, reducing the utilization of feed and protein synthesis, and thus inhibiting growth (42). It has been shown that the reduced growth performance of fish due to excess phenylalanine may be due to the energy consumption of excess phenylalanine in the body acting on deamidation, resulting in the oxidation of large amounts of phenyl pyruvic acid deposited in the body, producing toxic and even pathogenic effects (43). In Nile tilapia, it was shown that excess phenylalanine did not affect its growth performance (44). Other factors influenced by the cultural environment, such as water temperature, fish size, and amino acid composition, may also explain these disparities (45). It has also been suggested that the inhibitory effect of phenylalanine on fish growth may be because the body uses part of its energy to excrete nitrogen, which is because excess amino acids are easily degraded by the body and excreted in the form of nitrogen (46). However, the inhibitory effect of excess phenylalanine on fish growth is not conclusive, and more studies will be needed.

### Effects on the expression of genes related to growth in triploid *O. mykiss*

Intestinal health affects protein synthesis in the organism, which is regulated by TOR signaling molecules (47). When certain specific changes occur in the internal environment of the organism, the downstream effector protein S6K1 is regulated by TOR genes, thus participating in the regulation of cell growth, differentiation, and proliferation processes, while the downstream 4EBP-1 gene is also regulated by mTOR genes, regulating the growth process of the organism (48). When the S6K1 protein is activated in the cell, it phosphorylates several sites, including ribosomal protein S6, to promote the formation of the translation initiation complex (49). Silva found that IGF-1 is sensitive to changes in nutrients, especially amino acids (50). The relatively complex interaction between different hormones affects the growth regulation of hormones, among which GH, GHR, and IGF-1 are considered to be the most important growth-regulating genes. IGF-1 viability affects the secretion of growth hormones. The presence of growth hormone in the organism promotes the synthesis and release of this hormone, and the action of growth hormone on IGF-1 is mediated by the growth hormone receptor GHR, so GH-GHR binding is necessary to stimulate IGF-I synthesis and release (51). This study showed that the growth rate of triploid *O. mykiss* was slower when phenylalanine was deficient in the fish, but when phenylalanine was excessive, the WGR of *O. mykiss* had a more pronounced slowdown than when it was deficient. The expression of GH genes was highest at 2.29% phenylalanine level in the low fish meal diet, and the IGF-1 gene peaked at 2.03% and was significantly different from those of other groups. This has the same trend as the results obtained in Nile tilapia (52). The dietary amino acid imbalance was reported to reduce the expression level of the hepatic IGF-I gene in junco (*Rachycentron canadum*) (53) and Japanese seabass (*Lateolabrax japonicus*) (24). This is consistent with the findings of hybrid grouper larvae (*Epinephelus fuscointestinestatus*♀ × *Epinephelus lanceolatus*♂) (54), in which the treatment group with added complex protein had significantly higher daily feed intake and significantly higher rapamycin (TOR) liver target gene expression levels (55). In other amino acid studies, higher relative mRNA expression levels of rapamycin (TOR) and eukaryotic translation initiation factor 4E-binding protein (4E-BP) were observed in 17.5 and 15.0 g/kg Leu diets (56), In the study of valine on rainbow trout growth gene expression, TOR mRNA and elF4E binding protein (4E-BP) expression were observed to be higher at 18.0 g/kg Val (57), The most significant effect of leucine on TOR and 4E-BP mRNA gene expression levels in rainbow trout was 13.5 g/kg (58), the same trend as the results of this experiment. In conclusion, dietary phenylalanine had an improved effect on the expression of growth-related genes in triploid *O. mykiss* with low fish meal diets. However, for our study, not explaining our results at the protein level is a shortcoming, and we will do more studies in the future to explain this mechanism and explain it in the discussion.

### Effects of dietary phenylalanine levels on the antioxidant capacity of triploid *O. mykiss*

Phenylalanine is a specific amino acid containing a phenyl ring structure that binds to hydroxyl radicals and eliminates hydroxyl radicals as well as reactive oxygen species (ROS) from the muscle. Oxidative stress occurs when the production of excess ROS overwhelms the antioxidant defense system, leading to cytopathology (59). The main enzymes that have the role of oxidant scavengers are SOD, catalase, and glutathione peroxidase. Non-enzymatic antioxidants include glutathione and other thiol compounds (60). In this experiment, dietary phenylalanine levels reduced the MDA content and increased the SOD content in the liver, thereby inhibiting oxidative damage caused by lipids and proteins. A previous study showed that phenylalanine could inhibit lipid peroxidation and protein oxidation by reducing ROS production in fish gills and that the phenylalanine deficiency group had significantly reduced resistance to superoxide anions and hydroxyl radicals. It was suggested that this could be related to the fact that the phenyl ring of phenylalanine can combine with hydroxyl radicals to form three hydroxylation products that can have a positive effect on scavenging free radicals (61). It has also been reported that the effect of phenylalanine on SOD activity may be related to its ability to promote the release of dopamine, which enhances extracellular SOD protein expression and cell surface SOD activity in rat astrocytes (62). However, whether dietary phenylalanine can stimulate the release of dopamine in fish has not been studied. Similar results were obtained in the present experiments in *Pagrus major* (63) but the serum CAT levels did not differ significantly in this experiment in triploid *O. mykiss*, which may be due to the different sensitivity of different fish to the stimulation of CAT in the intestine.

### Effects of dietary phenylalanine levels on the digestion of triploid *O. mykiss*

The ability of fish to digest and absorb nutrients is closely related to the activity of intestinal digestive enzymes. Phenylalanine improves the digestive capacity of fish by promoting the growth of the pancreas and intestine, which in turn improves the secretion of digestive enzymes and thus the digestive level of fish (64). In this experiment, the addition of phenylalanine to low fish meal diets significantly increased the intestinal trypsin and amylase activities of triploid *O. mykiss*, both of which reached a maximum in the 2.03% treatment group but did not have a significant effect on lipase activity. In Nile tilapia, there were significant differences in lipase activity but no significant differences in amylase activity in the intestine, which may be due to differences in the location of the digestive enzyme assay (65). But how phenylalanine affects the secretion of intestinal digestive enzymes has not been studied. In the gibel carp study, phenylalanine significantly increased hepatopancreas weight, intestinal length, intestinal weight, intestinal fold height, hepatopancreas, and intestinal trypsin, chymotrypsin, amylase, and lipase activities in gibel carp, and had significant effects on digestion-related indices in gibel carp (66). In contrast to the present experiment, grass carp (22), as an herbivorous fish, has a different ability to digest lipids than that of triploid *O. mykiss*.

### Effects of phenylalanine levels on immunity-related indices in triploid *O. mykiss*

Fish may convert phenylalanine into tyrosine, which can then be turned into melanin and catecholamines, which are significant immunomodulators with immunomodulatory activities (67). To date, IL-1β findings have been published in many fish species, including grass carp (*C. idella*) (68), *O. mykiss* (69), European sea bass (*Dicentrarchus labrax*) (70), Atlantic salmon (*Salmo salar*) (71), Nile tilapia (72) and channel catfish (*Ictalurus punctatus*) (73). IL-6 was discovered in Japanese flounder (*Paralichthys olivaceus*) (74), *O. mykiss* (75), gilthead seabream (*Sparus aurata*) (76), bluntnose seabream (*Megalobrama amblycephala*) (77) and roughy (*Larimichthys crocea*) (78). IL-8 has been cloned and identified in many fish species. These include Atlantic cod (*Gadus morhua*) (79), *O. mykiss* (80), Japanese flounder (81), and zebrafish (*Brachydanio rerio*) (82). This study showed that the dietary phenylalanine to low fish meal diets had a positive effect on regulating the expression of genes related to intestinal immunity in triploid *O. mykiss*. The pro-inflammatory factors IL-2 and IL-6 reached minimal values in the 2.03 and 2.29% treatment groups and were significantly higher than in the control group. IL-1β reached minimal values in the 2.03% treatment group and was significantly lower than in the other treatment groups. The expression of IL-10, an anti-inflammatory factor, was highest in the 2.03 and 2.29% treatment groups and was significantly higher than in the other treatment groups. There are few reports on the effect of phenylalanine on intestinal inflammatory factors in fish. However, in humans, melanin can inhibit the production of cytokines such as IL-1β and IL-6 by human blood mononuclear cells because phenylalanine is a prerequisite for tyrosine, which can produce melanin, so we hypothesize that the gene expression of cytokines such as IL-1β, IL-6, and IL-2 in triploid *O. mykiss* is positively influenced by phenylalanine (83). Phenylalanine has been reported to reduce the number of peripheral blood lymphocytes in mice (*Mus musculus*). The production of peripheral blood lymphocytes in mice is stimulated by tetrahydrobiopterin, and phenylalanine promotes the production of tetrahydrobiopterin (84). Therefore, we speculate that phenylalanine also affects the expression of cytokines in triploid *O. mykiss* by affecting the number of its peripheral blood lymphocytes. However, the relevant studies on fish are few and need further validation.

### Effects of dietary phenylalanine levels on expression of tight junction protein-related genes in triploid *O. mykiss*

Fish intestinal health relies on a physical barrier composed of tightly linked proteins and epithelial cells. This study showed that either deficiency or excess of phenylalanine downregulated the expression of intestinal occludin, claudin-1, and ZO-1 in triploid *O. mykiss*. It has been shown that the function of the intestinal barrier is related to the inhibitory effect of phenylalanine on inflammatory factors. For instance, in human cells, IL-8 regulates the expression of occludin in vascular cells (85).

Tumor necrosis factor-α is also involved in tight junction protein expression regulation, which follows the same pattern as our experimental results. Lysine (86), arginine (87), methionine (88), and isoleucine (89) have all been studied for their effects on the expression of intestinal tight junction protein-related genes in fish, but less research has been done on phenylalanine. In grass carp, dietary phenylalanine could effectively improve the expression of intestinal tight junction proteins, with the highest expression of claudin-1, ZO-1, and occludin mRNA levels at 1.15% feeding (90). The expression of claudin-1, ZO-1, and occludin reached the highest values at a 2.03% phenylalanine level, which may be related to the different requirements of phenylalanine in the fish itself. As a result, adding appropriate phenylalanine to feed improves the regulation of tight junction protein expression in the organism and plays an important role in maintaining intestinal health.

## Conclusion

Dietary phenylalanine levels (2.03–2.64%) significantly increased the expression of intestinal growth-related genes and had a regulatory effect on the expression of immune-related genes in triploid *O. mykiss* fed a low fish meal diet (15%). Meanwhile, growth performance and body composition-related indicators have also been significantly improved. Using SGR and WGR as evaluation indices, the optimal requirement of phenylalanine for triploid *O. mykiss* was 2.13% by quadratic regression analysis. Based on the current research, the optimal phenylalanine addition level can be further explored to replace a fish meal with plant protein to provide a theoretical basis for the optimization of an artificial compound feed for triploid *O. mykiss.*

## Data availability statement

The original contributions presented in this study are included in the article/supplementary material, further inquiries can be directed to the corresponding authors.

## Ethics statement

The animal study was reviewed and approved by the Committee for the Welfare and Ethics of the Laboratory Animals of Heilongjiang River Fisheries Research Institute, CAFS. Written informed consent was obtained from the owners for the participation of their animals in this study.

## Author contributions

SZ completed the experiments and wrote the manuscript. CW, HL, and YY provided the experimental design and financial support. YW, SLiu, and HJ had key roles in the data processing and mapping processes. SH and SLu contributed to the test shop equipment and water quality control. All authors contributed to the article and approved the submitted version.
